# Myocardial fibrosis as a early cardiac marker of disease in patients with lamin A/C mutations

**DOI:** 10.1186/1532-429X-13-S1-O76

**Published:** 2011-02-02

**Authors:** Andrea Barison, Pier G Masci, Marianna Fontana, Matteo Milanesi, Roberta Poletti, Vincenzo Positano, Claudio Passino, Giovanni D Aquaro, Giancarlo Todiere, Michele Emdin, Massimo Lombardi

**Affiliations:** 1 Scuola Superiore Sant'Anna and Fondazione G Monasterio CNR - Regione Toscana, Pisa, Italy; 2 Fondazione G. Monasterio CNR - Regione Toscana, Pisa, Italy

## Objective

to assess myocardial fibrosis in lamin A/C mutations (LM) carriers using constrast-enhanced cardiac magnetic resonance (CMR).

## Background

LM carriers usually develop a dilated cardiomyopathy (DCM) phenotype in association with atrio-ventricular blocks and/or ventricular arrhythmias. However, sudden cardiac death may be the first clinical manifestation. Hence early detection of myocardial abnormalities before overt left ventricular (LV) dysfunction is warranted for a better risk stratification.

## Methods

Seventeen paucisymptomatic LM carries (age 41±16 years, 9 male), 14 paucisymptomatic DCM patients (age 59±11 years, 8 male, NYHA I-II) with mild LV dysfunction (ejection-fraction 40%-55%) and 12 healthy controls (age 46±10 years, 9 male) underwent complete clinical, echocardiographic, biohumoral and contrast-enhanced CMR assessment. Cine CMR was used to derive LV volumes, mass and function, and post-contrast CMR to detect late gadolinium enhancement (LGE) as an index of gross fibrosis. Eight LM patients, all DCM patients and all normal controls underwent pre- and post-contrast myocardial T1 mapping with gadolinium partition coefficient calculation, as an index of interstitial fibrosis.

## Results

LM carriers presented a similar LV end-diastolic volume (85±17 ml/m2, p=NS) and slightly reduced ejection-fraction (61±6% p<0.001) than controls (83±19 ml/m2, 67±6%), while in DCM patients LV end-diastolic volume (102±17 ml/m2) and ejection-fraction (49±5%) were worse than both controls and LM carriers (p<0.001). Moreover, DCM patients presented worse diastolic dysfunction (9±3 vs 7±1, p<0.01), NT-proBNP plasma levels (438±416 vs 108±139 ng/l, p<0.01) and Doppler-estimated pulmonary pressure (33±7 vs 30±5 mmHg, p<0.01) than LM carriers. Six (43%) DCM patients presented DE, representing 9±3% of LV mass; similarly, seven (42%) LM carriers had DE, but with a larger extension (11±5% of LV mass, p<0.03). In LM carries DE distribution was patchy (2 pts) or midwall (5 pt), similarly to DCM patients (patchy in 1 pt, midwall in 4 pts, subendocardial in 1 pt). GPC reached a steady state 5 minutes after gadolinium injection. In DCM patients GPC was slightly higher than controls (0.42±0.05 vs 0.38±0.06, p=0.04), while in LM carriers it was much higher (0.45±0.07) than controls (p<0.01). Even excluding hyperenhanced myocardium, in LM carriers GPC remained higher than controls, while in DCM patients only slightly higher than controls.

## Conclusions

Myocardial fibrosis occurs early in LM carrier before LV dilatation and dysfunction and may represent an early phenotypic expression of cardiac involvement.

